# 

**Published:** 1868-02

**Authors:** 


### Books and Pamphlets Received.

On the Signs and Diseases of Pregnancy. By Thomas Hawks Tanner, M. D., F. L. S., Member of the Royal College of Physicians, etc. From the second and enlarged London edition; with four colored plates and illustrations on wood. Philadelphia: Henry C. Lea. 1868. Breed, Lent & Co.

Annual Abstract of Therapeutics, Materia Medica, Pharmacy and Toxicology, for 1867; followed by an Original Memoir on Gout, Gravel and Urinary Calculi. By A. Bouchardat, Professor of Hygiene, in the Faculty of Medicine, Paris. Translated and edited by J. M. De Rosset, M. D. Adjunct to the Professor of Chemistry, University of Maryland. Philadelphia: P. Lindsay & Blakisten.

